# Antihypertensive and Renal Protective Effects of Oryeongsan in Spontaneously Hypertensive Rats

**DOI:** 10.1155/2020/8844031

**Published:** 2020-12-21

**Authors:** Kiwan Kang, Minjeong Jeong, Hongjun Kim, Beomjin Lim, Sangjun Kim, Insoo Jang

**Affiliations:** ^1^Department of Internal Medicine, College of Korean Medicine, Woosuk University, Jeonju 54987, Republic of Korea; ^2^Department of Pediatrics, College of Korean Medicine, Woosuk University, Jeonju, Jeonbuk 54987, Republic of Korea; ^3^Department of Prescription, College of Korean Medicine, Woosuk University, Jeonju, Jeonbuk 54986, Republic of Korea; ^4^Department of Pathology, Yonsei University College of Medicine, Seoul 03722, Republic of Korea; ^5^Jeonju AgroBio-Materials Institute, Jeonju, Jeonbuk 54810, Republic of Korea

## Abstract

Oryeongsan (ORS), a traditional medicine used to regulate body fluids, has a long history of use as a diuretic in Korea, China, and Japan. ORS is commonly thought to lower blood pressure, but high-quality data on its effects are sparse. The purpose of this study was to determine the antihypertensive and renal protective effects of ORS in rats with hypertension. Spontaneously hypertensive rats (SHR) were divided into two groups with similar mean baseline systolic blood pressure (SBP) and diastolic blood pressure (DBP). Then, 10 mL/kg of vehicle (distilled water) or 200 mg/kg of ORS extract were administered orally once a day for 3 weeks. SBP and DBP were measured at weeks 1, 2, and 3. At the end of the experiment, blood was collected, and kidneys were removed for histology. By the 2nd and 3rd week after initiation of treatment, the ORS-treated group had significantly lower SBP than control-treated rats (191.3 ± 6.5 vs. 206.3 ± 9.8 mmHg, *p* = 0.022 at the 2nd week; 195.8 ± 7.8 vs. 217.0 ± 8.1 mmHg, *p* = 0.003 at the 3rd week, respectively). The ORS-treated group trended toward having a lower DBP than control, but there was no significant difference. Blood urea nitrogen (BUN) and serum creatinine (Cr) were not different between the ORS-treated and control groups (BUN: 23.7 ± 1.1 vs. 22.7 ± 2.8 mg/dL, *p* = 0.508; Cr: 19.0 ± 2.2 vs. 21.6 ± 2.1 *μ*M, *p* = 0.083, respectively). The percentage of renal tissue affected by tubulointerstitial fibrosis was significantly lower in the ORS-treated group (1.68 ± 0.60) compared to controls (3.17 ± 0.96, *p* = 0.019). These findings suggest that treatment with ORS reduces SBP and ameliorates renal damage in SHR.

## 1. Introduction

Hypertension is a leading risk factor of death from cardiovascular and chronic kidney disease. Indeed, lowering blood pressure is the most effective way to prevent stroke and slow the progression of hypertensive renal disease [1]. Despite decades of intensive efforts to design and implement methods to control blood pressure, 46% of US adults have hypertension [2]. Globally, 31.1% of adults had hypertension, and the prevalence of hypertension is expected to continue to rise [3]. Diuretics are one of the oldest and safest classes of antihypertensive drugs. Thiazide diuretics have been used to treat hypertension for about 60 years [4] and are associated with fewer side effects than other antihypertensive agents [5]. The average drop in blood pressure (BP) in studies involving thiazide diuretics was 14.5/6.7 mmHg (systolic/diastolic) [6]. As a result, they are still recommended as first-line agents by the European Society of Cardiology, the American Society of Hypertension, and the Eighth Joint National Committee [7]. Oryeongsan (ORS), a traditional medicine used to regulate body fluids, has a long history of use as a diuretic in Korea, China, and Japan [8]. In animal experiments, ORS increases urine volume and sodium excretion [9]. Generally, all classes of diuretics have the effect of lowering blood pressure by decreasing blood volume [10]. Thus, it is predicted that ORS influence on blood pressure, but there are not many high-quality studies using ORS for hypertension [11, 12]. There have been no reports on the effect of ORS in SHR, except for one in China [13], and the goal of this research is to investigate the effects of ORS on hypertension and kidney damage in spontaneous hypertensive rats (SHR).

## 2. Materials and Methods

### 2.1. Plant Materials and Preparation of ORS

The medicinal herbs required to formulate ORS were purchased from Kwangmyungdang Medicinal Herbs Co. in Korea and authenticated by the Department of Prescription, College of Korean Medicine, Woosuk University. The formula of ORS was based on the Chinese Pharmacopoeia [8], and the composition ratio is listed in Table 1. First, 1.6 kg of ORS was extracted with 16 L of 30% ethanol at room temperature (25°C) for 24 hours. The ethanol extract was then filtered through filter paper no. 2 (Advantec, Japan) and concentrated using a rotary evaporator (Eyela rotary evaporator NE-2001, Japan) at 60°C. Finally, 225 g of ORS extract was obtained by freeze-drying the concentrated ethanol extract (Ilshin freeze dryer FD8508, Korea) at −75°C. The constituents of ORS were subsequently analyzed by the chromatographic fingerprint (see Supplementary Materials).

### 2.2. Animals and Experimental Protocol

This study was conducted in compliance with Good Laboratory Practice (GLP) regulations and was approved by the KPC Labs Institutional Animal Care and Use Committee (protocol P172005). Male spontaneously hypertensive rats (SHR/NCrlCrlj, SHR, 8 weeks old) were purchased from Charles River Laboratories Japan, Inc. (Yokohama, Japan). The rats were individually housed in standard laboratory cages at standard temperature (21–24°C), humidity (45–60%), and lighting conditions (12 h light/dark) with free access to rodent chow and water.

When they were 10 weeks old, the rats were distributed into two groups according to systolic blood pressure (SBP) and diastolic blood pressure (DBP), creating a similar average baseline blood pressure in both groups (Table 2). Each treatment group had five rats. Rats were treated with 10 mL/kg vehicle (distilled water) or 200 mg/kg ORS extract (at 20 mg/mL) prepared by the method described above. Vehicle and ORS extract were administered via oral gavage once a day for 21 days.

At the end of 3 weeks, the rats were anesthetized with isoflurane, and 10 mL blood was collected from the abdominal aorta (10 mL SST tube, Vacutainer, BD, USA). The right kidneys were removed, and one slice of the middle transverse section from each kidney was fixed in 10% buffered formalin solution and then embedded in paraffin. The remaining portion of each kidney was stored at −70°C until protein extraction.

### 2.3. Blood Pressure Measurement

Blood pressure was measured on the 7th, 14th, and 21st day after the start of treatment. Measurements of SBP and DBP were repeated three times for each animal, and the mean value was recorded. The animals were allowed to rest for at least 15 minutes at 30°C before blood pressure was measured. Blood pressure was measured by a tail-cuff using a BP-2000 series II instrument (Visitech Systems, USA).

### 2.4. Hematological and Serum Biochemical Analysis

Blood samples were centrifuged for 10 minutes at 6,000 rpm at 4°C. Blood urea nitrogen (BUN) and creatinine (Cr) were measured using DRI-CHEM-4000i (Fujifilm Co., Japan).

### 2.5. Histology

Paraffin-embedded kidneys were sectioned into 4-*μ*m thick sections and then stained with periodic acid-Schiff and trichrome according to common methods. Glomerular injury was measured by determining the sclerosis index [14]. Every glomerulus in each kidney section was scored according to the extent of sclerosis (0, no sclerosis; 1, sclerosis of <25% of the glomerulus; 2, 25–50%; 3, 50–75%; 4, >75%), and the average of these scores was considered the final sclerosis index for each kidney section. Tubulointerstitial injury was evaluated by morphometric analysis using ImageJ software (Version 1.51j8, National Institutes of Health, Bethesda, MD, USA). Ten consecutive digital images at 200x magnification were captured within the cortex of each kidney. Images containing large arteries were replaced with other images because periarterial fibrosis might affect subsequent analysis. Software was used to quantify the fibrotic area (bright blue in color after the trichrome stain), and the fibrotic area percentage was calculated for each kidney.

### 2.6. Statistical Analysis

Data collected during the study were expressed as mean ± SD. Statistical analysis was performed with independent *t*-tests using SPSS 26.0 (IBM Co., USA). Comparisons resulting in *p* values <0.05 were considered significant.

## 3. Results

### 3.1. Effect of ORS on Blood Pressure

**Figure 1:**
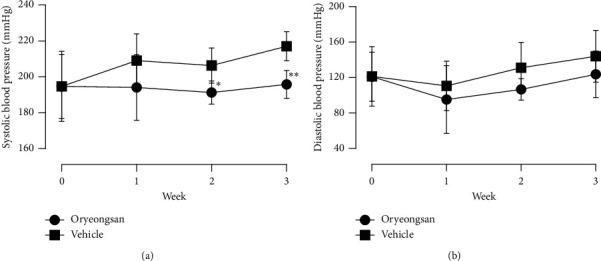
Changes in systolic and diastolic blood pressure of spontaneously hypertensive rats (SHR) treated with oryeongsan or vehicle control for 3 weeks. Data are expressed as mean ± SD. ^*∗*^*p* < 0.05 and ^*∗∗*^*p* < 0.01 compared with vehicle.

At the beginning of treatment, the average SBP in the ORS-treated group and vehicle-treated-control group (control) was similar (194.7 ± 19.6 vs. 194.7 ± 17.9 mmHg, respectively). One week after treatment, the ORS-treated group showed a nonsignificant trend toward a lower SBP (194.1 ± 18.3 mmHg) relative to control (209.0 ± 7.8 mmHg). On the 2nd and 3rd week after treatment, the ORS-treated group had significantly lower SBP than control (191.3 ± 6.5 vs. 206.3 ± 9.8 mmHg, respectively, *p* = 0.022 at 2nd week; 195.8 ± 7.8 vs. 217.0 ± 8.1 mmHg, respectively, *p* = 0.003 at 3rd week). During the 2nd and 3rd week, the ORS-treated group showed a trend toward lower DBP than the control group, but there was no significant difference (Figure 1 and Table 2).

### 3.2. Effect of Oryeongsan on Renal Function

**Figure 2:**
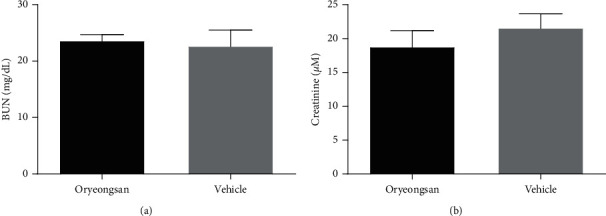
Serum levels of blood urea nitrogen (BUN) and creatinine (Cr) at the time of sacrifice. There was no significant difference between the oryeongsan-treated and vehicle-treated groups.

BUN and serum Cr at the time of sacrifice were not different between the ORS-treated group and the control group (BUN: 23.7 ± 1.1 vs. 22.7 ± 2.8 mg/dL, respectively, *p* = 0.508; Cr: 19.0 ± 2.2 vs. 21.6 ± 2.1 *μ*M, respectively, *p* = 0.083) (Figure 2).

### 3.3. Effect of ORS on the Development of Glomerulosclerosis and Tubulointerstitial Fibrosis

**Figure 3:**
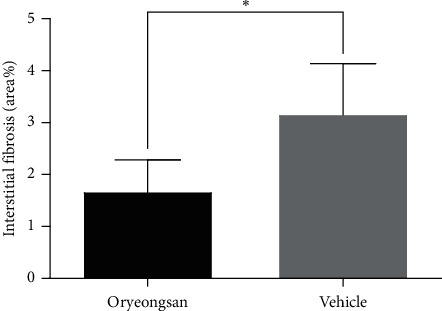
Morphometric analysis of tubulointerstitial fibrosis in the kidney of spontaneous hypertensive rats (SHR) treated with oryeongsan or vehicle. Data are presented as mean ± SD. ^*∗*^*p* < 0.05 compared with vehicle.

**Figure 4:**
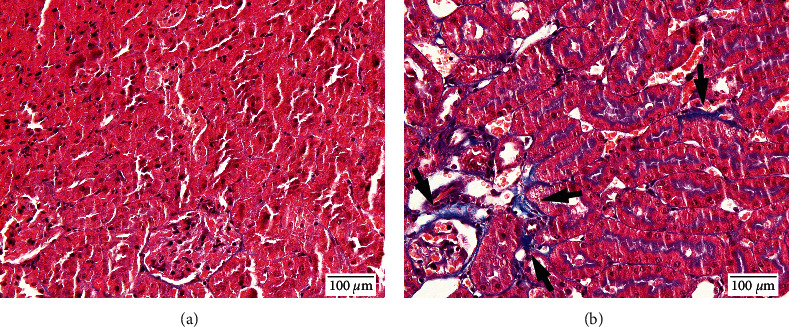
Representative kidney samples of spontaneously hypertensive rats (SHR) treated with oryeongsan (a) and vehicle (b). The fibrotic area of the renal cortex is stained in bright blue color (arrows).

The glomerular sclerosis index was generally very low in both groups (0.00 ± 0.0 in ORS-treated group vs. 0.01 ± 0.01 in control, *p* = 0.127) meaning that, in this animal model, hypertension did not cause significant injury. However, the percentage of tissue area with evidence of tubulointerstitial fibrosis was significantly smaller in the ORS-treated group (1.68 ± 0.60) than in the control group (3.17 ± 0.96, *p* = 0.019) (Figures 3 and 4).

## 4. Discussion

ORS is composed of five medicinal herbs, each of which is known to have a diuretic or antihypertensive effect. In each animal experiment, ethyl acetate and *n*-butanol, the major components of *A. orientale* and *P. cocos*, were found to increase urine volume and electrolyte excretion [15, 16]. In addition, *P. umbellatus* extract increased urine volume and electrolyte excretion by inhibiting the mRNA expression of aquaporin 2 and vasopressin 2 receptors, which are involved in resorption of water in the kidney [17]. *A. macrocephala* also exhibited a diuretic effect by inhibiting the mRNA expression of aquaporin 2 [18]. Cinnamaldehyde, a major component of *C. cassia*, is related to blood pressure regulation, and it exhibited a vasodilatory effect by inhibiting the influx and release of Ca^+^ through an endothelial independent pathway [19]. Studies using ORS in hypertension models have been conducted in China and Japan. In stroke-prone spontaneously hypertensive rats (SPSHR), feed mixed with ORS was given to rats for 8 weeks. ORS showed diuretic effect by lowering the concentration of vasopressin in urine, but did not show a significant change in urine volume and blood pressure. However, since ORS used in this experiment did not follow the composition ratio of the original prescription, there were limitations to confirming the diuretic and antihypertensive effects of ORS [20]. In the 2 kidney 1 clip (2K1C) model, rats were orally administered with 20–80 g/kg of ORS for 30 days every day, and an increase in urine volume and a decrease in SBP were observed in all ORS-treated groups [21]. SHRs are a well-established model of human essential hypertension and are characterized by a progressive increase of blood pressure with age [22]. In the present study, the ORS-treated group had significantly lower SBP than control, suggesting that ORS helped manage blood pressure in SHR. In previous studies in China, animals treated daily with 4.8–19.2 g/kg of ORS for 8 weeks had significantly lower SBP compared to control animals [13]. The dose of ORS used in this study was 200 mg/kg, indicating that a lower dose of ORS is also effective in lowering blood pressure. The mechanism by which ORS lowers blood pressure is currently unclear. In recent studies, ORS has been shown to be associated with the renin-angiotensin-aldosterone system (RAAS) [23]. In one study, ORS decreased plasma renin activity and aldosterone concentrations and induced diuresis by inhibiting the RAAS [9]. On the other hand, it has been shown that ORS promotes diuresis by blocking the sodium channel of the distal tubule in a manner similar to thiazide diuretics [24]. Thus, the antihypertensive effects of ORS seem to be related to diuretic action, but further research is needed to fully elucidate the mechanism. Serum BUN and Cr levels of SHR were not different in ORS-treated mice even after 3 weeks of daily treatment. This means that even if the reduction in blood pressure occurred due to diuretic activity, there was likely no loss of renal function due to dehydration. In studies conducted on hyperuricemic and diabetic mice, treatment with ORS lowered serum BUN and Cr levels and showed a renal protective effect [25, 26]. Hypertensive nephrosclerosis is caused by damage to the renal parenchyma due to high blood pressure and is typically associated with pathohistological changes in the renal vessels, glomeruli, and interstitial tissues [27]. The evaluation of renal damage requires visual examination and computerized morphological analysis by a trained pathologist [28]. Although glomerulosclerosis was not observed in this study, tubulointerstitial fibrosis was significantly inhibited in the ORS-treated group. Tubulointerstitial fibrosis is a condition of clinical concern because it leads to chronic renal failure in most kidney diseases [14]. The renal protective effects of ORS have been confirmed in other studies, which have shown that it suppresses the expansion of the mesangial matrix in diabetic rats [26, 29].

## 5. Conclusions

Together, our findings suggest that ORS is effective in reducing SBP and ameliorating renal damage in SHR. Furthermore, these studies provide new evidence supporting the renal protective effects of ORS in SHR. One main limitation of this study was that we could not determine whether ORS acted via a diuretic effect. Future research should clarify the mechanisms by which ORS lowers blood pressure and should directly compare its effects against other diuretic agents.

## Figures and Tables

**Table 1:** Formulation for oryeongsan.

Herbal name	Scientific name	Weight ratio	Origin
Alismatis Rhizoma	*Alisma orientale* Juzepzuk	5.0	Korea
Poria Sclerotium	*Poria cocos* Wolf	3.0	China
Atractylodis Rhizoma	*Atractylodes macrocephala* Koidzumi	3.0	China
Polyporus	*Polyporus umbellatus* Fries	3.0	China
Cinnamomi Cortex	*Cinnamomum cassia* Presl	2.0	Vietnam

**Table 2:** Blood pressure of spontaneously hypertensive rats (SHR) treated with oryeongsan or vehicle control for the experimental period.

	SBP (mmHg)				DBP (mmHg)			
	Baseline	Week 1	Week 2	Week 3	Baseline	Week 1	Week 2	Week 3
Oryeongsan (*n* = 5)	194.7 ± 19.6	194.1 ± 18.3	191.3 ± 6.5^*∗*^	195.8 ± 7.8^*∗∗*^	120.9 ± 27.7	95.2 ± 38.2	106.6 ± 12.2	123.6 ± 26.2
Vehicle (*n* = 5)	194.7 ± 17.9	209.0 ± 14.9	206.3 ± 9.8	217.0 ± 8.1	121.3 ± 33.5	110.6 ± 27.9	131.1 ± 28.7	143.9 ± 29.2

The results are expressed as mean ± SD. ^*∗*^*p* < 0.05; ^*∗∗*^*p* < 0.01 compared to the values of vehicle-treated SHR. SBP, systolic blood pressure. DPB, diastolic blood pressure.

## Data Availability

The experimental data used to support the findings of this study are available from the corresponding author upon request.

## Supplementary Materials

Supplementary Materials Figure S1: chemical structures of the representative compounds for chemical profiling by HPLC. Table S1: contents (mg/kg) of markers in processed herbal medicine extracts by HPLC. Figure S2: comparative chromatograms on markers of cinnamic acid (1), cinnamic aldehyde (2), alisol B 23-acetate (3), and atractylenoide III (4) in the standard solution (A∼C) and oryeongsan extracts (D) by HPLC.
