# Supplementary material for: Antihypertensive and Renal Protective Effects of Oryeongsan in Spontaneously Hypertensive Rats
Source: Evid Based Complement Alternat Med. 2020 Dec 21;2020:8844031. doi: 10.1155/2020/8844031 (PMC7803283; doi:10.1155/2020/8844031)
Supplement: Supplementary Materials — Figure S1: chemical structures of the representative compounds for chemical profiling by HPLC. Table S1: contents (mg/kg) of markers in processed herbal medicine extracts by HPLC. Figure S2: comparative chromatograms on markers of cinnamic acid (1), cinnamic aldehyde (2), alisol B 23-acetate (3), and atractylenoide III (4) in the standard solution (A∼C) and oryeongsan extracts (D) by HPLC. [file 8844031.f1.docx]

# Supplementary marterials

**Chemical profiling of markers from Oryeongsan extracts by HPLC**

Oryeongsan is a herbal medicine complex, and consists of Alismatis Rhizoma (*Alisma oriental*), Poria Sclerotium (*Poria cocos*), Atractylodis Rhizoma (*Atractylodes macrocephala*), Polyporus (*Polyporus umbellatus*), and Cinnamomi Cortex (*Cinnamomum cassia*). As the reference standard of ORS marterials, trans-cinnamic acid and cinnamic aldehyde (*C. cassia*), alisol B 23-acetate (*A. orientale*) and atractylenoid III (*A. macrocephala*) were selected. The structural formula for each component of the reference standard is shown in figure S1 (Figure S1).


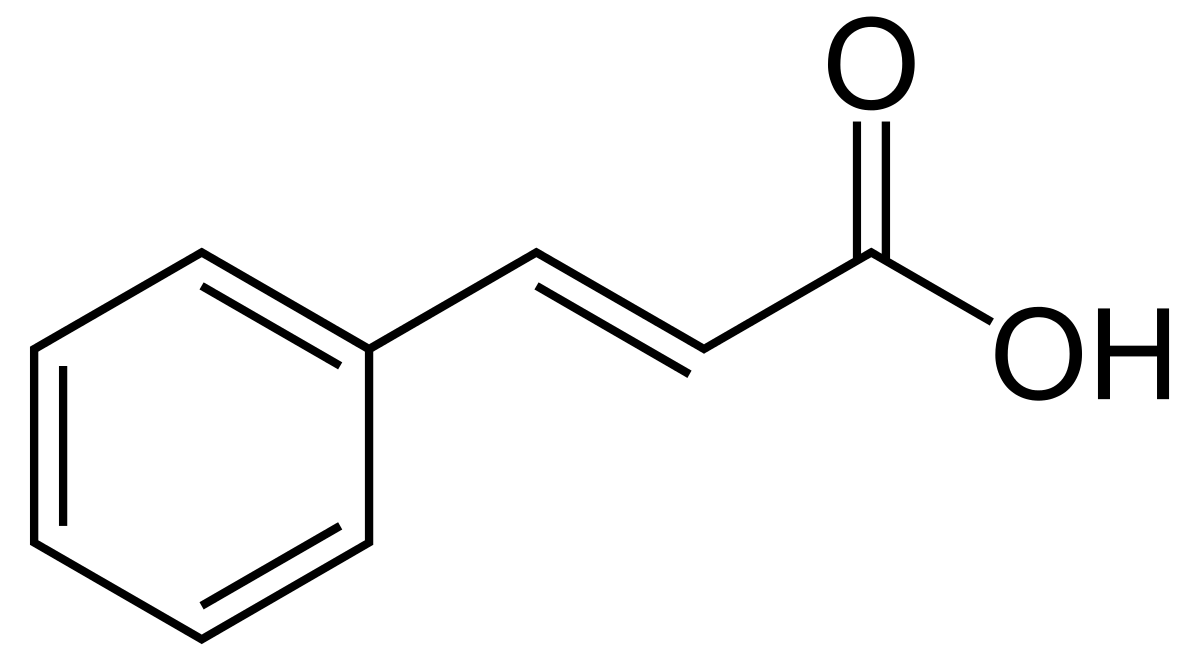

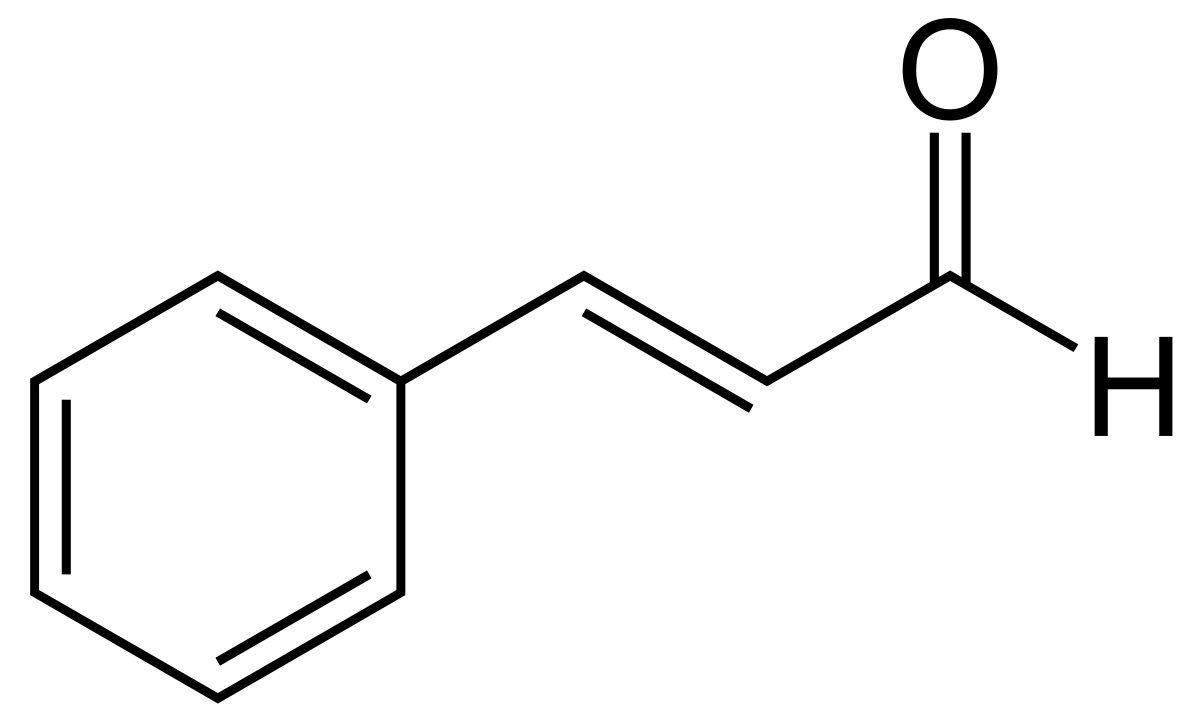


trans-Cinnamic acid (1) Cinnamic aldehyde (2)


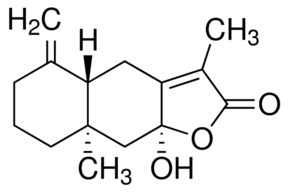

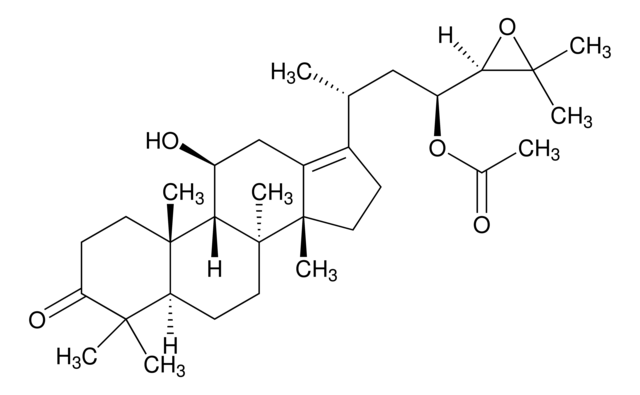


Atractylenoid III (3) Alisol B 23-acetate (4)

Figure S1. Chemical structures of the representative compounds for chemical profiling by HPLC.

The High Pressure Liquid Chromatography (HPLC) of the ethanol extract of ORS was analyzed by Agilent 1200 series with MWD (Multi-Wavelength Detector) (Agilent, USA) equipped with UV detector (254–280 nm). The chromatographic separation was conducted on Aegispak-L C18 column (4.6 × 200 mm, 3 μm pore size, YoungJin Biochrom, Korea) at 35°C with a flow rate of 0.5 mL/min and injection volume of 15 μL. The mobile phase was composed of 0.1% Aqeous formic acid / acetonitrile (A/B, v/v) as a gradient elution: 0 min-10% B, 13 min-10% B, 20 min-25% B, 24 min-30% B, 28 min-35% B, 32 min-45% B, 35 min-45% B, 40 min-50% B, 43 min-55% B, 47 min-60% B, 50 min-60% B, 55 min-10% B. All concentrations used in this test were indicated as free base.
 After quantitative evaluation using HPLC, the content of each raw material extract was shown in below (Table S1, Figure S2).

Table S1. Contents (mg/kg) of markers in processed herbal medicine extracts by HPLC.

| Sample information | Chromatographic results (µg/kg)1) | | | |
| --- | --- | --- | --- | --- |
|  | Cinnamic acid | Cinnamic aldehyde | Alisol B-23 acetate | Atractylenoid III |
| Oryeongsan | 23.58 | Not detected | 37.02 | 449.37 |

1) Each standard compound was presented as the free form.

Figure S2. Comparative chromatograms on markers of cinnamic acid (1), cinnamic aldehyde (2), alisol B 23-acetate (3) and atractylenoide III (4) in the standard solution (A ~ C) and Oryengsan extracts (D) by HPLC.
